# Nanoporous Microsponge Particles (NMP) of Polysaccharides as Universal Carriers for Biomolecules Delivery

**DOI:** 10.3390/nano10061075

**Published:** 2020-05-31

**Authors:** Maria Federica Caso, Felicia Carotenuto, Paolo Di Nardo, Alberto Migliore, Ana Aguilera, Cruz Matilde Lopez, Mariano Venanzi, Francesca Cavalieri, Antonio Rinaldi

**Affiliations:** 1 NANOFABER srl, 00123 Rome, Italy; maria.federica.caso@gmail.com; 2 Center of Regenerative Medicine, University of Rome “Tor Vergata”, 00133 Rome, Italy; carotenuto@med.uniroma2.it (F.C.); dinardo@uniroma2.it (P.D.N.); 3 Department Clinical Sciences and Translational Medicine, University of Rome “Tor Vergata”, 00133 Rome, Italy; 4 I.M. Sechenov First Moscow State Medical University, 119991 Moscow, Russia; 5 Rheumatology Department, San Pietro Hospital Fatebenefratelli, 00189 Rome, Italy; migliore.alberto60@gmail.com; 6 Center for Genetic Engineering and Biotechnology, Havana 10600, Cuba; ana.aguilera@cigb.edu.cu (A.A.); matilde.lopez@cigb.edu.cu (C.M.L.); 7 Department of Chemical Science and Technologies, University of Rome “Tor Vergata”, 00133 Rome, Italy; venanzi@uniroma2.it; 8 PROMAS-MATPRO Laboratory, Sustainability Department, ENEA, 00123, Rome, Italy

**Keywords:** drug delivery, hyaluronic acid, alginate, dextran, CM-dextran, slow delivery

## Abstract

Different polysaccharides—namely dextran, carboxymethyl dextran, alginate, and hyaluronic acid—were compared for the synthesis of nanoporous microsponges particles (NMPs) obtained from a one-pot self-precipitation/cross-linking process. The morphologies and sizes of the NMPs were evaluated comparatively with respect to polymer-to-polymer and cross-linker solvents (water-based vs. DMSO). We found that the radial distribution of the polymer in the near-spherical NMPs was found to peak either at the core or in the corona of the particle, depending both on the specific polymer or the solvent used for the formation of NMPs. The NMP porosity and the swelling capability were evaluated via scanning electron microscopy (SEM). The degradation study indicated that after 10 h incubation with a reducing agent, approximately 80% of the NMPs were disassembled into soluble polysaccharide chains. The adsorption and release capacity of each type of NMP were evaluated using fluorescently labeled bovine serum albumin and lysozyme as model proteins, highlighting a release time typically much longer than the corresponding adsorption time. The dependence of the adsorption-release performance on pH was demonstrated as well. Confocal microscopy images allowed us to probe the different distribution of labeled proteins inside the NMP. The safety and non-cytotoxicity of NMPs were evaluated after incubation with fibroblast 3T3 cells and showed that all types of NMPs did not adversely affect the cell viability for concentrations up to 2.25 μg/mL and an exposure time up to 120 h. Confocal microscopy imaging revealed also the effective interaction between NMPs and fibroblast 3T3 cells. Overall, this study describes a rapid, versatile, and facile approach for preparing a universal non-toxic, nanoporous carrier for protein delivery under physiological conditions.

## 1. Introduction

Organic–inorganic nanoporous multifaceted structures, such as micro-nanoflowers [1], microsponges [2], nanoclews [3], and nanococoons [4] are emerging materials that have proven useful in applications spanning catalysis, sensing, and drug delivery, owing to their porous structure. The fabrication of these systems typically requires the addition of an inorganic component such as a metal salt (e.g., composed of copper, calcium, or manganese phosphate) to biomacromolecules (e.g., peptide [5], DNA [6], RNA, or polysaccharides [7]). This approach involves the functional groups of the biomacromolecules, which provide binding sites for the nucleation and subsequent growth of metal phosphate crystals, leading to the formation of nanostructured phosphate-based microparticles, with a high surface-to-volume ratio and signature porous structures. Such nanoporous microparticles have primarily been used for the immobilization of enzymes with preserved catalytic performances [8], for intracellular imaging, and for protein and drug delivery [9]. For instance, bioactive proteins were recently encapsulated into DNA hybrid flowers. It was demonstrated that the DNA flowers could deliver payloads of cytotoxic protein (i.e., RNase A) to the cells without a loss in its biological function and structural integrity, resulting in highly increased cell death compared to the free protein [10]. Compared to alternative methods for fabricating nanoporous microparticles, which include hydrothermal/solvothermal synthesis, thermal decomposition, suspension, emulsion and precipitation polymerization [11], multiple emulsion–solvent evaporation, and inorganic template methods [12], the micro-nanoflowers synthesis is a relatively facile and reproducible approach. As it does not require any toxic elements or extreme harsh conditions, it was developed to overcome the limitations of conventional methods.

The further development of this method for engineering multifunctional nanoporous microparticles under mild conditions is therefore desirable. Instead of using metal salts, a new co-precipitation method was recently developed by our group to use an organic molecule, i.e., cross-linking agent, to obtain polysaccharides nanoporous microsponge particles (NMPs) without resorting to metal ions [13]. During the microparticles preparation, the polysaccharide chains present in the reaction mixture mediate the nucleation and growth of cross-linking agent crystals leading to NMP formation with signature structural and functional properties. Compared to the inorganic salts used in the synthesis of micro-nanoflowers, the developed cross-linker offers several advantages including reactivity with amine hydroxyl and carboxyl groups and redox responsive behavior. We recently reported the application of this approach to engineer biodegradable and biocompatible NMPs made of high molecular weight hyaluronic acid. Therein, we demonstrated that the hyaluronic acid NMPs exhibit a sustained release of the polysaccharide under both redox and enzymatic treatment. After injection into an intra-articular body cavity of healthy mice, these particles can reside at the point-of-delivery for over five weeks, slowly releasing hyaluronic acid without exerting any local or systemic toxic side effect [14]. Such findings encourage the deployment of this biomedical technology for the sustained delivery of hyaluronic acid. In addition, NMPs hold greater potential for regenerative medicine and pharma-cosmetics as a drug delivery system for synthetic drugs and biomacromolecules in general (e.g., peptides, growth factors, mRNA, etc.). Applications of NMPs in the food industry could be possible as well and might be explored in the future.

To that end, in this work, we seek to demonstrate the potential use of the developed cross-linked NMP formulation as a universal template to synthesize nanoporous particles from different polysaccharide building blocks while proving safety and protein loading capability. Specifically, we demonstrate the general use of our technique to prepare porous polysaccharide NMPs from dextran, carboxymethyl (CM) dextran, alginate, and low molecular weight hyaluronic acid. Notably, our simple methodology greatly expands the selection of diverse polysaccharides to be used as templates of the nanoporous microparticles found in the literature. The potential exploitation of the NMP as a platform for protein delivery is extremely high and—as a proof of concept—we report the successful loading of NMPs with two proteins of scientific and pharmacological relevance, i.e., bovine serum albumin (BSA) and lysozyme. Because these proteins have different molecular weights and isoelectric points, they represent significant case studies to assess the loading and release capacity of the polysaccharides NMPs.

NMPs provide a platform for delivery a variety of biomacromolecules, such as proteins, lipids, and nucleic acids which can hardly pass through the various biological barriers without the assistance of a carrier. Polysaccharides are widely regarded and studied as potential carrier materials for site specific drug delivery because of their non-toxic, biocompatible and biodegradable properties [15,16,17,18]. These systems can be used to provide targeted delivery of drugs (at the cellular or tissue level) and, for example, to improve their ophthalmic or oral bioavailability [19], as well as in the intra-articular therapy of chronic rheumatic disease. Preliminary biological studies on cell-NMPs interactions indicate possible applications of NMPs as universal carriers of biomolecules to be furthered in subsequent work.

## 2. Materials and Methods

### 2.1. Materials

Cystamine dihydrochloride (CYS), 1.1 Carbonyldiimidazole (CDI), sodium alginate (Alginate, MW determined by viscosimetry was 140 kDa), dextran sulfate sodium salt (Dextran, MW 40 kDa), carboxymethyl-dextran sodium salt (CM-Dextran-MW 70 kDa), hyaluronic acid (low MW HA, 20–70 kDa), Rhodamine B isothiocyanate (RITC), DL-Dithiothreitol 99% (DTT), bovine serum albumin, 96% (BSA), dimethyl sulfoxide (DMSO), and lysozyme from chicken egg white, 90% were purchased from Sigma-Aldrich (St. Louis, MO, USA).

### 2.2. Synthesis and Characterization of the Cross-Linking Solutions

The cross-linker (CL) solution was obtained from CDI and CYS dissolved in DMSO in a 3:2 weight ratio. The reaction proceeded overnight without stirring. Alternatively, another cross-linker solution was obtained by dissolving CDI and CYS in MilliQ water to obtain a precipitate. Subsequently the precipitate was dissolved in MiliQ at pH 4 by adding hydrochloric acid 2M (as needed). NMR spectra were recorded in deuterated DMSO to characterize the cross-linker (NMR spectrometer operating at 400 MHz, Bruker DRX, Bruker AVANCE, Billerica, MA, USA).

### 2.3. Synthesis and Characterization of NMP

One-milliliter samples of 1% *w/v* solution of dextran, CM-Dextran, hyaluronic acid, and alginate were individually added to cross-linker solutions without stirring. The resulting NMP particles were centrifuged and washed with MilliQ water three times. The yields of the reactions were calculated as follows: the NMP were formed using fluorescently labeled polysaccharide and the UV-vis absorbance “ab” (Cary 100 spectrophotometer, Agilent, Santa Clara, CA, USA) of the polysaccharide solution was measured before NMP microparticles formation. Next the suspension was centrifuged and the supernatant analyzed by measuring the UV-vis absorbance “aa”. The yield was estimated by comparing the two absorbances as “(ab − aa)/ab 100%”.

Particles were characterized with a Zeiss fluorescence microscope (Axio Scope.A1) equipped with a mercury-vapor short-arc-lamp HB = 50 W/AC at 560 nm emission wavelength and 63 × objective, a confocal microscope (FluoView, 1000 LSCM system, Olympus, Tokyo, Japan), and a SEM (model Leo Supra-35, Carl Zeiss Microscopy GmbH, Jena, Germany). Prior to SEM observation, the samples were vacuum-dried. The above procedure applied identically for polymer derivatives labeled with RITC (ref. Section 2.4). SEM and confocal microscopy images were used to measure the diameters of NMP in vacuum-dry and wet conditions respectively. The comparison between the two measurements was used to estimate the swelling ratio. Pore size distribution of NMP was evaluated using SEM micrographs of vacuum-dried NMPs. Particle diameter and pore size measurements were carried out on micrographs of about 100 different microparticles using ImageJ (NIH software, Version 1.51, Bethesda, MD, USA).

### 2.4. Preparation of Polysaccharide Fluorescently Labeled with Rhodamine B Isothiocyanate

For NMPs used in fluorescence assays, the standard preparation of polysaccharide in Section 2.3 was modified by dissolving Rhodamine B isothiocyanate (RITC) into DMSO and by adding it to the standard 1% solution of polysaccharide. The resulting solution was stirred for a few hours at room temperature. To remove residual non-conjugated dyes, the samples were dialyzed using a dialysis membrane 12–14,000 Daltons (Medicell International, London, UK), and the degree of functionalization were calculated using the absorbance measured for pre-weighted samples of stained polysaccharide and the Beer–Lambert–Bouguer law.

### 2.5. Degradation Kinetics of NMP

The degradation study was carried out over 24 h when the degradation appeared complete. An aliquot of 1 mL was used. At variable time intervals, the suspension was centrifuged, and the supernatant was analyzed using UV-vis spectroscopy. The supernatant contains the microparticles components after disassembly including the fluorescently labeled polysaccharides. The debris was not analyzed using SEM.

### 2.6. Protein Adsorption and Release in Particles

Lysozyme and BSA were labeled as follows: Rhodamine B isothiocyanate (RITC) was dissolved into DMSO and 10 uL were added to 1 mg/mL protein solution. The resulting solution was stirred for a few hours at room temperature. To remove residual non-conjugated dyes, the samples were dialyzed using a dialysis membrane 12–14,000 Daltons (Medicell International, London, UK); the degree of functionalization was calculated as described in Section 2.3.

The loading of the proteins was performed as follows: 1.75 mg of RITC-lysozyme and 1.75 mg of RITC-BSA were dissolved in 1 mL PBS at pH 7 (precisely pH 7.4). Next, a suspension of NMP containing 1 mg of polysaccharides was added to the protein solutions. At variable intervals, the resulting suspensions were centrifuged, and the supernatants were analyzed using UV-vis spectroscopy (peaks at 560 nm for RITC). The plateau in the adsorption curve was obtained after 24 h incubation up to 250 h and the loading capacity was calculated using the maximum absorption values in the adsorption curves (@24 h plateau).

To study dependence of protein loading and unloading in NMPs as a function of pH solution, an additional 1.75 mg of RITC-lysozyme (lysozyme from chicken egg white, Sigma-Aldrich, St. Louis, MO, USA, 90%) and 1.75 mg of RITC-BSA (bovine serum albumin, Sigma-Aldrich, St. Louis, MO, USA, 96%) were solved in 1 mL of PBS at pH 5 and 11 to obtain 4 more solutions. Each solution was monitored during 24 h at room temperature to test the stability of proteins at the different pH levels. d-NMPs and w-NMPs (polysaccharides concentration in both: 1.5 g/L) obtained from CL dissolved in DMSO and in H2O respectively were added to the NMPs, yielding 48 different samples for each combination of {protein (2), polysaccharide (4), CL protocol (2), pH (3)}. At variable intervals, the resulting suspensions were centrifuged, and the supernatants were analyzed with UV-vis spectroscopy (peaks at 560 nm for RITC). The plateau of adsorption curve—that is, the maximum of adsorption—was measured after 24 h. The lysozyme and BSA adsorbed particles were centrifuged and suspended in 1 mL of PBS at pH 5, 7, and 11. The resulting suspensions were centrifuged at variable intervals and the supernatant was analyzed with UV-vis spectroscopy. Every 24 h, fresh PBS was added. The plateau of release curve was obtained at 150 h for the sake of calculation (but the study was carried out for 300 h).

### 2.7. Cell Assays for Cito-Toxicity and Adhesion/Internalization of NMPs

Fibroblasts 3T3 were chosen as the model cell line and cultured at 37 °C in a 5% CO_2_ atmosphere, in DMEM and L-Glutamine supplemented with 10% bovine calf serum (Gibco, Waltham, MA, USA). Cells were seeded at a density of 5 × 10^3^ cells/cm^2^ (on 24-well plates or cover slips) and were allowed to attach for 24 h prior to the experiments until the population confluence reached approximately 80%. The NMPs were premixed with culture medium and incubated with 3T3 fibroblasts for different times and concentrations.

#### 2.7.1. Cell Viability

The trypan blue was used to determine the number of viable cells incubated with particles. It is based on the principle that live cells possess intact cell membranes that exclude certain dyes such as trypan blue, whereas dead cells do not [20]. The cells were cultured to 80% confluence in 24-well plates and each type of NMP was added to medium for 24 or 120 h in individual wells. The 120 h time point was selected arbitrarily to show the impact of NMP on cell viability at prolonged incubation time. Cells cultured without NMPs were considered as controls. In each set of experiments, the polysaccharide concentration was considered. An additional control was performed by adding the CL in complete medium without the polysaccharide. After incubation for 24 and 120 h, the medium was removed and collected, and the cells were detached using 0.25% Trypsin/EDTA solution. Following this step, the cell suspension was centrifuged, and the cells were stained with 0.4% tryptan blue (Sigma-Aldrich, St. Louis, MO, USA). The stained cells were visualized with an inverted microscope (Leica, Wetzlar, Germany). The dead and living cells were counted using a Bürker-Türk counting chamber and the percentage of living cells was determined.

#### 2.7.2. Study of Cell Adhesion/Internalization of NMPs by Confocal Microscopy

To investigate at the confocal microscope the possible internalization of NMP microsponges by 3T3 fibroblasts, NMPs were labeled with fluorescein-5-isothiocyanate (FITC). After culturing the cells for 24 h, they were incubated in plates with FITC-labelled NMP particles (0.25 g/mL) in DMEM 10% serum for 4 h, 24 h, or 48 h. At the end of each time period, the cells were washed with PBS to remove the excess/unbound NMPs and dead cells. The dead and living cells were counted using a Bürker-Türk counting chamber and the percentage of living cells was determined as an estimate of adherent cells after 4 h, 24 h, or 48 h incubation with NMPs. After incubation, cells were fixed and stained with a red fluorescent marker wheat germ agglutinin (WGA) conjugated to Texas Red (10 μg/mL; Sigma-Aldrich) for 15 min at 37 °C to label the cell, which selectively recognizes plasma and Golgi/ER membrane structures. Afterwards, the cells were fixed with cold methanol and stained with Hoescht 33258 (1 μg/mL; Sigma-Aldrich) to visualize the nuclei. Such samples were observed by means of a confocal laser scanning microscopy (Olympus Fluoview 1000, Tokyo, Japan). Confocal X-Y, X-Z, and Y-Z sections in max intensity projection mode (MIP modality) were taken to display three-dimensional images of MPs cell interaction.

## 3. Results and Discussion

### 3.1. Two-Ways Parametric Study on NMP Synthesis vs. (i) Polymer and (ii) Cross- Linker Solvent

Eight batches (4 × 2) of nanoporous polysaccharide microsponges particles (NMP) were synthetized according to our standard one-pot self-precipitation/cross-linking method [13,14] using four RITC-labeled polymers (i.e., alginate, hyaluronic acid, CM-dextran and dextran) and two alternative protocols based on solvent selection (i.e., DMSO vs. H_2_O route). The purpose was to investigate the effects on structure and morphology of the resulting NMPs for a given polymer–solvent pair. The results are summarized in Table 1, which displays for each batch the representative micrographs obtained via (i) confocal microscopy and (ii) scanning electron microscopy. The latter techniques provide complementary information and, while SEM reveals the overall morphology (e.g., shape, dimension, surface roughness, porosity), confocal microscopy clearly renders an insight about the “structural” properties in terms of polymer density within the NMP. The morphologies of the microsponges obtained in the DMSO—water mixture (d-NMP) and in pure water (w-NMP)—demonstrate the successful formation of NMP with all different polysaccharides and indicate that CL can also react with either neutral polysaccharides or negatively charged polysaccharides chains to form NMPs. The cross-linker crystalline domains form the scaffold of the particles, whereas the polymer chains act as a “glue” to bind the CL crystalline domains together. Yet, the kinetics of cross-linking and crystallization processes must be carefully controlled to obtain NMP. In fact, as a negative example, the procedure failed with chitosan and did not yield NMPs.

**Figure 1:**
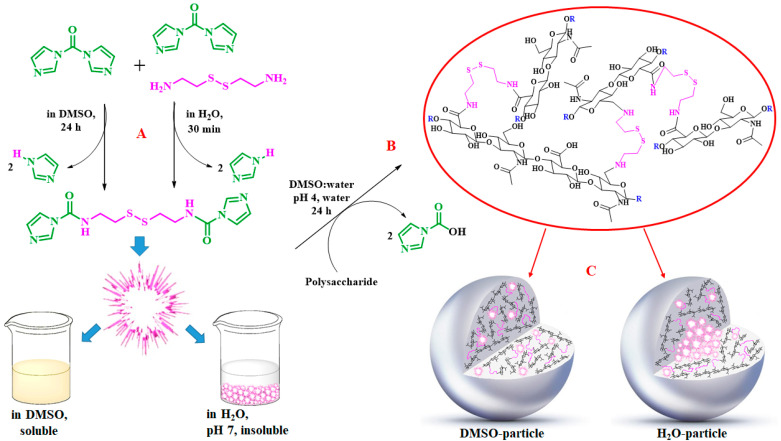
Scheme of preparation of nanoporous polysaccharide microparticles. (**A**) The cross-linker can be prepared in DMSO or MilliQ water; (**B**) The carbonyl imidazole moiety activates either the carboxylic groups or hydroxyl groups of the polysaccharides to form amide or ester cross-linkages. (**C**) The polysaccharide chains serve as the backbone for nucleation and crystallization of the cross-linking agent (different densities reflect observations from confocal microscopy). Note that CDI catalyzes the reactions and does not enter the final NMP.

Remarkably, the w-NMPs consistently exhibit a core shell structure with a less fluorescent core and a highly fluorescent corona. This indicates that the RITC-polysaccharides are predominately located on the periphery of the NMP. Conversely, the d-NMPs synthetized via the DMSO route shows an even distribution of the fluorescent polysaccharides throughout the NMP for dextran and HA and a fluorescent core in the case of alginate and CM-dextran. These different structures can be ascribed to the different kinetic of crystallization of the CL in water and DMSO. The faster precipitation of the CL in water produces a crystalline core excluding the polysaccharides chains; subsequently, the polysaccharides chains form a composite polysaccharides-CL porous outer corona. During the slow precipitation of CL in the case of DMSO water solvent, the polysaccharide chains remain embedded during the entire process of growth of NMP. A peculiar onion-like structure was observed for alginate-based d-NMP with an inner layer composed primarily of CL. The schematics in Figure 1 illustrate the general NMP formation process, highlighting that the carbonyl imidazole moieties present on the CL can react with either the carboxylic groups or hydroxyl groups of polysaccharides to form amide or ester cross-linkages respectively. The structure of the reactive molecule of the CL was confirmed using NMR spectra and mass spectroscopy (Supplementary Materials).

**Figure 2:**
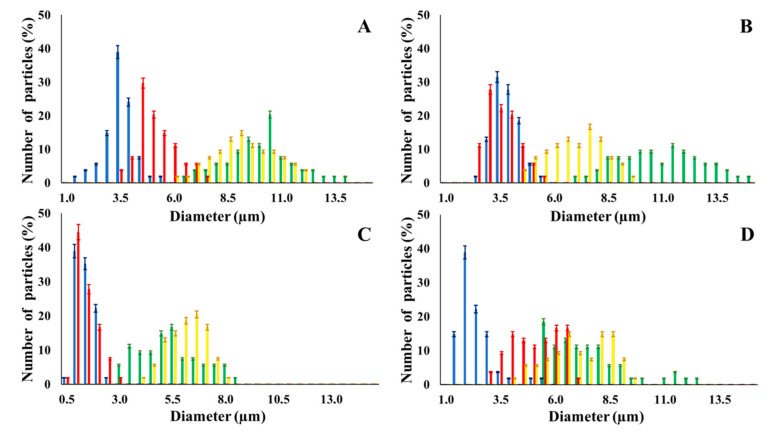
NMP diameters. Blue: dry d-NMP; green: swollen d-NMP; red: dry w-NMP; yellow: swollen w-NMP. (**A**) CM-dextran; (**B**) Hyaluronic Acid; (**C**) Alginate; (**D**) Dextran.

Table 2 summarizes some relevant outcomes of the synthesis process (i.e., NMP diameter, surface pore size, yield, swelling ratio) for each batch in this analysis. The NMPs always show a highly porous outer surface with pores ranging from 60 to 160 nm as estimated from SEM images in the vacuum-dry state. As for the NMP diameter, SEM and confocal analysis deliver information about swollen and dry particles, allowing the computation of the swelling ratio of NMPs in water. The results suggest that the particles are quite uniform in size, either in dry or swollen state. The high swelling ratio values measured for any NMP ranged from 1.6 to 5.3 and indicated a high capability to adsorb water in all cases. The swelling capability of NMP is a telling ex-post measurement of capillary suction of a NMP microsponge, which is very relevant for drug upload, and is correlated to both the nanoporosity structure and the elastic response of the CL system. In particular, a discernible difference in the porosity was noticed for alginate-based NMPs, exhibiting a remarkable capacity to swell up to 500% in combination with the smaller pores. For drug delivery purposes, the optimal pore diameter (indicative of the capillarity structure for a given NMP diameter) could be seen as a trade-off between maximizing the effective water uptake and ensuring sufficient higher cut-off in the size of drugs or biomacromolecules to enter into the NMP. For a better comparison of NMP diameter results, the size distributions for each batch are shown in Figure 2. As a final consideration, in this paper, we purposely used a much lower molecular weight hyaluronic acid (MW = 20–70 kDa) in comparison to our prior report (MW > 600 kDa), to verify the robustness of the fabrication method and the difference in NMP diameter, which decreases for smaller MW.

From a synthesis standpoint, the reaction yields for each polysaccharide are consistently quite high, which is industrially relevant when considering that such values were observed also while scaling up the reaction.

Finally, the degradation of NMP under redox treatment was evaluated as it is an important parameter for applications entailing the slow release of the polysaccharide. As the CL bears a disulfide bridge, we verified the ability of NMP to be deconstructed under redox treatment, similarly to what was done in our prior work [14]. RITC labeled NMPs were incubated in a solution of DTT 10 mM in PBS a reducing agent that could mimic the glutathione action in the biological environment. After 10 h incubation with DTT, approximately 80% of NMPs were disassembled into soluble polysaccharide chains (NMP degradation curve in Supplementary Materials).

### 3.2. NMP as Platform for Sequestration and Release of Proteins.

**Figure 3:**
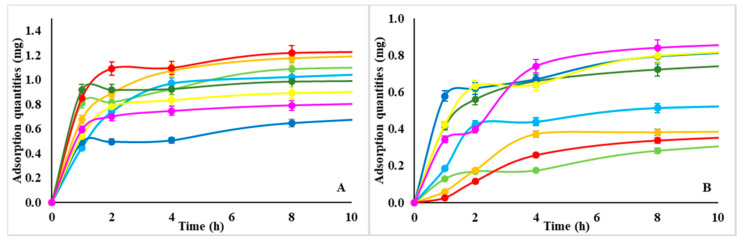
NMP adsorption trend—adsorbed milligrams of protein by d-NMP (**A**) and w-NMP (**B**). **Lysozyme:** CM-dextran in light green, hyaluronic acid in light blue, alginate in orange, and dextran in red. **BSA:** CM-dextran in dark green, hyaluronic acid in blue, alginate in yellow, and dextran in pink.

To demonstrate the marked capability of NMPs to sequestrate proteins from a medium at physiological conditions (pH 7.4), we chose BSA and Lysozyme as two models of positively and negatively charged proteins respectively. In addition, while BSA is a relatively large protein (MW 65 kDa) and an isoelectric point (pI) of 5.3, Lysozyme is a small enzyme (MW 14 kDa) with a pI of 11.4. Non-labeled NMPs were incubated in aqueous solutions of RITC labeled proteins for 24 h; the adsorption capacity was quantified after the centrifugation of NMP and analysis of the supernatant (Table 3). The adsorption capacity of each NMP was evaluated when a plateau in the absorption curve was reached (Figure 3).

**Figure 4:**
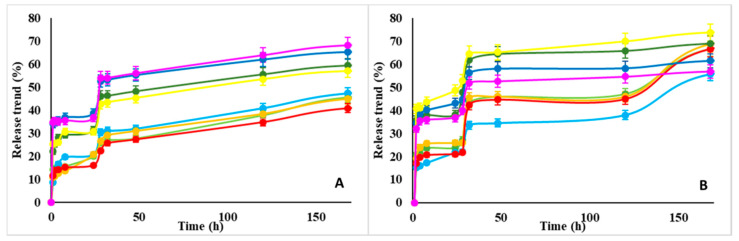
NMP desorption trend—Protein percent release by d-NMP (plot ((**A**)) and w-NMP (plot ((**B**)). **Lysozyme:** CM-dextran in light green, hyaluronic acid in light blue, alginate in orange, dextran in red. **BSA:** CM-dextran in dark green, hyaluronic acid in blue, alginate in yellow, dextran in pink.

The release of proteins from loaded NMPs was evaluated next by replacing PBS solution and monitoring release via UV-vis spectroscopy at different incubation times up to 180 h. The release curves of all NMPs are shown in Figure 4 Regardless, the different distribution of the proteins inside the NMP the release curves showed a step-wise profile with two steps. The first step was completed within 24 h incubation with the medium, whereas the second step exhibited a burst followed by a prolonged and sustained release trend of proteins from NMPs lasting up to 250 h. Such a burst in release may be ascribed the loosely bound proteins on the NMP outer surface.

The comparison between loading and release efficiency for Lysozyme and BSA in Figure 3 and Figure 4 indicates the following.

1. Proteins uptake of d-NMP and w-NMP varies between 0.8–1.2 and 0.4–0.8 mg protein/mg polysaccharide, respectively. Lysozyme is preferentially taken up by d-NMP, whereas serum albumin is equally adsorbed by both d-NMP and w-NMP. This indicates that the composite nanostructured template of d-NMP interacts more efficiently with different type of proteins.
2. On the other end, the efficiency of w-NMP (70–80%) in releasing the adsorbed proteins is higher than d-NMP efficiency (40–70%).
3. The adsorption is nearly completed after 4 h while the release typically occurs on a scale of days.

Overall these results demonstrate the capability of NMPs towards uploading proteins and highlight a characteristic asymmetry between adsorption and desorption. In fact, the strong capillary interactions that are responsible for the efficient protein-loading of NMPs is also the cause of slow release during discharge, opposing the osmotic gradient pull.

#### Dependence of Adsorption/Release Profiles vs. pH and Protein Distribution within the NMP

The release profile depends on a number of parameters, and one of the most important of which for many biomedical applications (e.g., drug delivery and intra-articular slow delivery) is the pH encountered by loaded NMP. Therefore, in addition to the above results related to physiological condition (PBS at pH 7.4, hereafter shortened as pH 7), we designed an experiment to assess the dependence of both adsorption and release performance of the eight NMP batches at pH 5 and 11. Hence, the six PBS solutions at pH 5, 7, and 11 containing RITC-lysozyme or RITC-BSA (6 samples) were monitored for 24 h at room temperature to ensure the stability of proteins loading and release.

**Figure 5:**
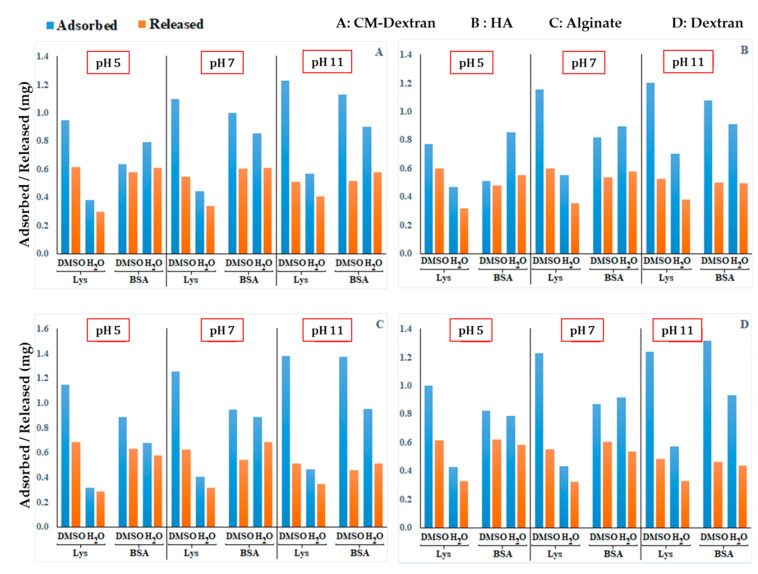
Adsorption (at 24 h—blue bar) and release (at 150 h—red bar) yields vs. pH 5, 7, and 11 for all 48 samples in the combinatorial study for each combination of factors (levels) {protein (2), polysaccharide (4), CL protocol (2), pH (3)}. Four subfigures refer to: (**A**) CM-Dextran, (**B**) Hyaluronic Acid, (**C**) Alginate, (**D**) Dextran.

**Figure 6:**
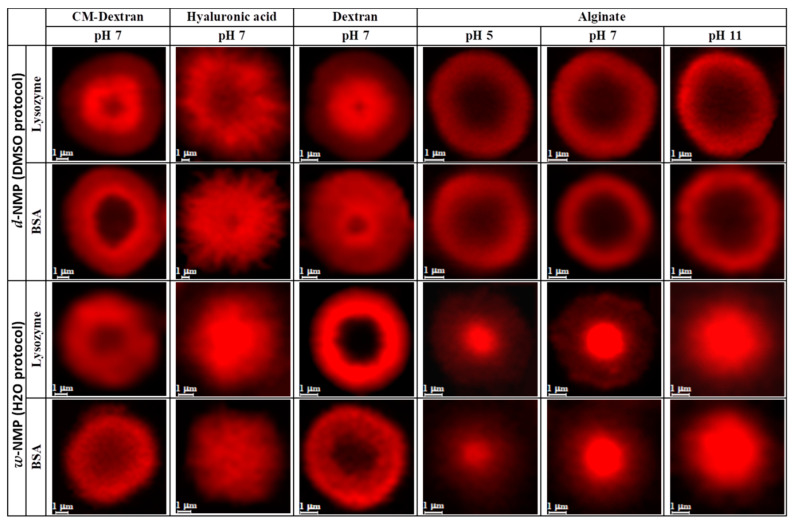
Confocal micrographs of RITC labeled lysozyme and BSA adsorbed by NMPs at pH 7. For alginate, pH 5 and 11 are also shown to highlight strong dependence of adsorption on pH value.

By the adsorption and desorption procedure described above, the measurement of the two plateau of the adsorption curve (i.e., the maximum of adsorption at 24 h) and of release curve (after 150 h) rendered the results in Figure 5 for each of sample in the experiment. The general findings emerging from this study are as follows:

1. The absolute adsorption/release performance depends on the specific NMP polysaccharide (i.e., size, porosity etc., as discussed in Section 3.1);
2. The adsorption and release rates tend to increase with the pH value (also Figure 6), although this effect is more marked on lysozyme than BSA.

The actual uptake of the protein, apart from the gross value, depends also on how it penetrates and distribute within the NMP, which varies considerably between the 48 cases. Observations from confocal microscopy help appreciating this aspect by rendering the distribution of (RITC-labelled) protein content within each NMP type (Figure 6).

Overall, these results indicate that the NMP nanostructures are endowed with a nanoporous morphology and high swelling capacity, which allows them to potentially accommodate bio-therapeutics such as proteins, DNA, and RNA, as well as small solutes dissolved in the up-taken aqueous phase. The protein adsorption inside the NMP is driven by a number of factors, including for example, (i) porosity and permeability of the composite template, (ii) hydrophobic interaction with the CL crystalline domain, and (iii) electrostatic interactions with the negatively charged polysaccharides. These factors are intertwined and dominate the overall distribution of the proteins inside the NMP template. Each NMP system shows a peculiar distribution of the proteins that is highly correlated to the specific and unique nanostructure of each NMP instance. Yet, we may try to better rationalize underlying (multi-factors) trends by using advanced statistical tools in future work. For instance, alginate-based d-NMP adsorb both proteins on the outer layer precluding their access in the core. Conversely alginate-based w-NMP more efficiently accommodate proteins in the hydrophobic core with pH dependence, indicating that the sequestration of proteins by alginate NMP can be controlled in different physiological environments such as the stomach (acidic) or the gut (basic).

### 3.3. The Interaction of NMP with Fibroblast T3T Cells: Assessing Toxicity and Adhesion/Internalization by Cells

#### 3.3.1. Toxicity Assays

**Figure 7:**
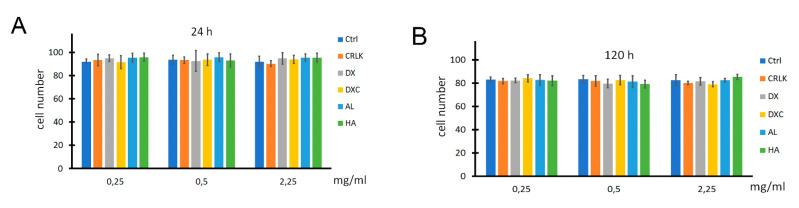
In vitro cytotoxicity of the NMPs vs fibroblasts 3T3. Cell viability after 24 h (**A**) and 120 h (**B**) for NMP and cross linker (CRLK) concentrations of 0.25, 0.5, and 2.25 mg/mL. The Ctrl is “control” consisting of plain cell culture.

The toxicity of NMPs was assessed on T3T fibroblasts as survival cells in trypan blue test under several experimental conditions. Cells were grown in complete medium (Ctrl) or in complete medium supplemented with different concentrations ranging from 0.25 mg/mL to 2.25 mg/mL of all eight batches of NMPs, for 24 and 120 h. As the CL exerts a vital role in the fabrication of NMPs, an additional control (CRLK) was performed by adding the CL in complete medium without the polysaccharide. Results are reported in Figure 7 as percentage of living cells (*n* = 3, *p* < 0.05). No value was found significant compared to the relative controls, indicating lack of cytotoxicity of both CL and NMPs. This confirms that NMPs are indeed safe and non-toxic, as observed in our prior study [14]. These results support a possible use of NMPs as excipient, as well as for extracellular or intracellular release of different type of cargos.

#### 3.3.2. Study of Cell Interaction and Uptake of NMPs

**Figure 8:**
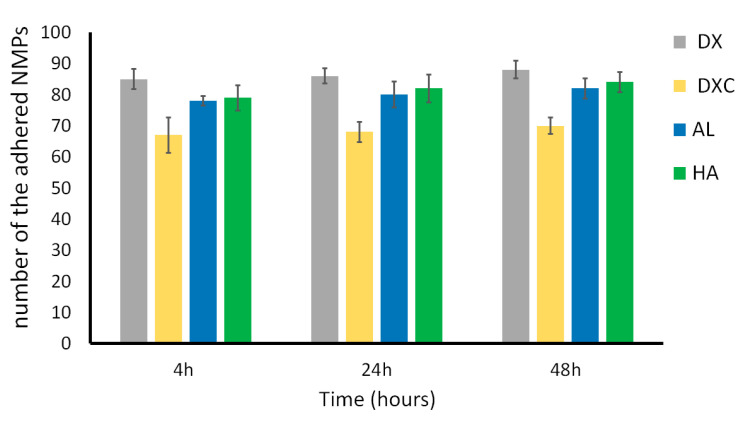
NMP cellular uptake by cells cultured with FITC-labeled NMPs in mw24 plates for 4 h, 24 h, or 48 h. Free NMPs counted via Bürker–Türk counting chamber. Results are presented as number of adhered NMPs over the NMP number added to the medium at time 0.

To analyze the interaction between NMPs to 3T3 cells, FITC-labelled NMPs were added to fibroblasts cultured in 24 multiwells. The number of NMPs in each sample was quantified after 4, 24, and 48 h incubation by counting via a Bürker– Türk counting chamber with the unbound NMPs removed from suspension. The results showed that a large fraction of NMPs readily associated with fibroblast within the first 4 h of incubation (Figure 8). However associate NMPs can be either bound to the cell membrane or internalized by endocytic processes.

**Figure 9:**
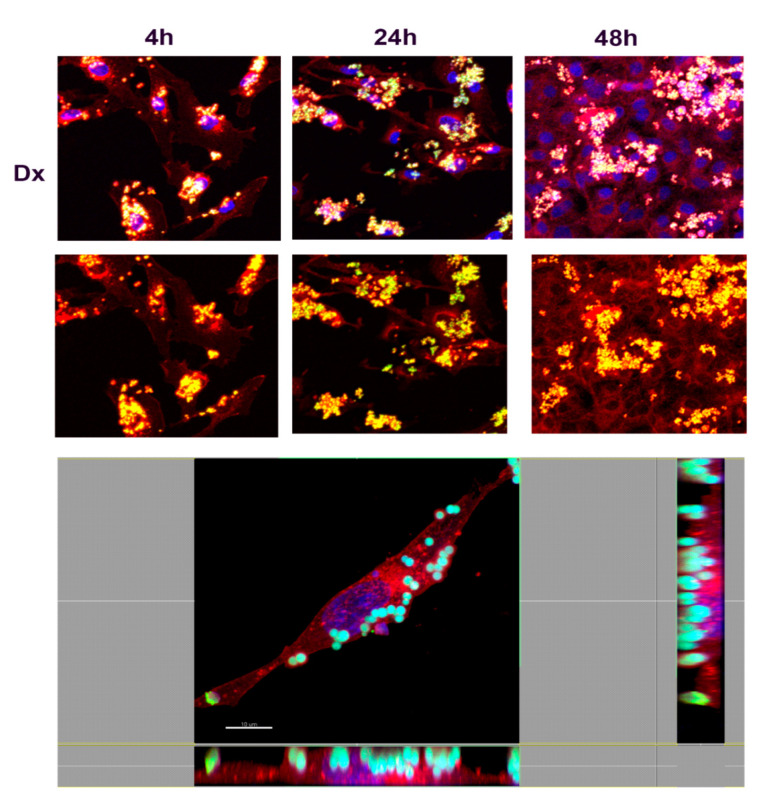
Confocal laser scanning microscopy images of dextran NMP cellular uptake. (TOP panel) Representative images of fibroblasts treated with FTIC-labeled dextran (DX) NMPs (green) for indicated time (4 h, 24 h, and 48 h). The cells were stained with WGA conjugated to Texas Red (red) and counterstained with Hoescht 33258 to visualize the nucleus (blue). Scale bars, 30 µm. (BOTTOM panel) Confocal X-Y, X-Z, and Y-Z sections in max intensity projection mode (MIP modality). Side panels present *z*-sections along the *x* and *y* planes and show the 3D distribution of cells, confirming that DX-NMPs are clearly attached to the cell on the outside of the membrane after 4 h incubation. Scale Bar, 10 µm.

**Figure 10:**
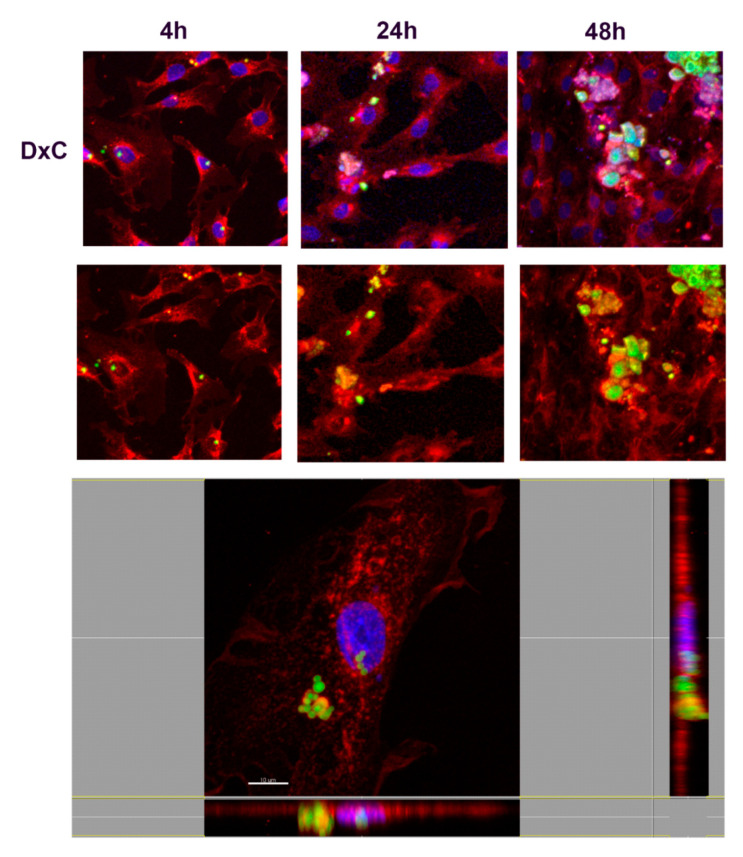
Confocal laser scanning microscopy images of carboxylate dextran (DxC) NMP cellular uptake. (TOP panel) The cells were stained with wheat germ agglutinin conjugated to Texas Red (red) and counterstained with Hoescht 33258 to visualize the nucleus (blue). Scale bars, 30 µm. (BOTTOM panel) Confocal X-Y, X-Z, and Y-Z sections in max intensity projection mode (MIP modality). Side panels present *z*-sections along the *x* and *y* planes and show the 3D distribution of cells, confirming that DXC-NMPs were present on the cell, attached on the outside of the membrane after 4 h incubation. Scale Bar, 10 µm.

**Figure 11:**
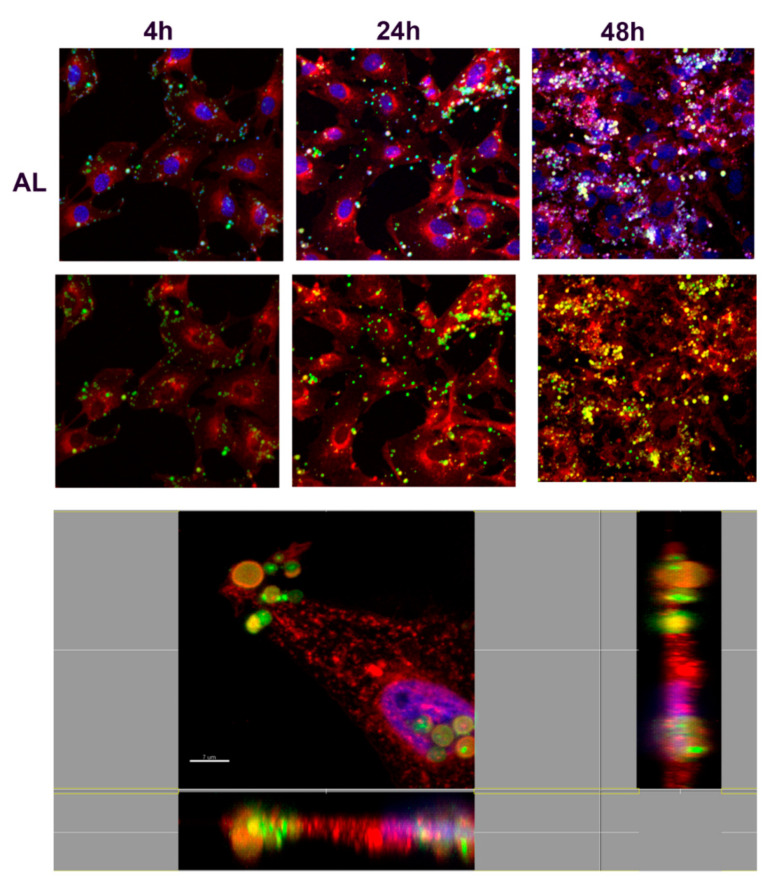
Confocal laser scanning microscopy images of alginate NMP cellular uptake. (TOP panel) Representative images of fibroblasts treated with FTIC-labeled alginate (AL) NMPs (green) for indicated time (4 h, 24 h, and 48 h). The cells were stained with wheat germ agglutinin conjugated to Texas Red (red) and counterstained with Hoescht 33258 to visualize the nucleus (blue). Scale bars, 30 µm. (BOTTOM panel) Confocal X-Y, X-Z, and Y-Z sections in max intensity projection mode (MIP modality). Side panels present *z*-sections along the *x* and *y* planes and show the 3D distribution of cells, confirming that AL-NMPs were present in the cell surrounding after 4 h incubation. It is not clear in this case whether the NMPs are on the outside or on the inside of the cell. Scale Bar, 7 µm.

**Figure 12:**
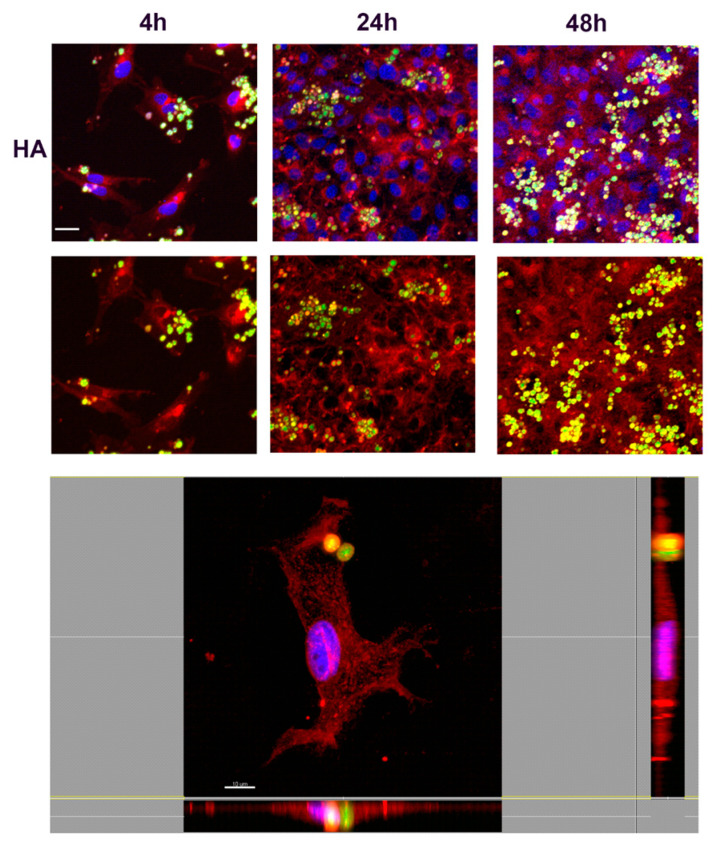
Confocal laser scanning microscopy images of hyaluronic acid (HA) NMP cellular uptake. (TOP panel) The cells were stained with wheat germ agglutinin conjugated to Texas Red (red) and counterstained with Hoescht 33258 to visualize the nucleus (blue). Scale bars, 30 µm. (BOTTOM panel) Confocal X-Y, X-Z, and Y-Z sections in max intensity projection mode (MIP modality). Side panels present *z*-sections along the *x* and *y* planes and show the 3D distribution of cells, confirming that HA-NMPs were attached on the outside of the cell membrane after 4 h incubation. Scale Bar, 10 µm.

In order to understand the nature of the uptake and determine whether NMPs localized inside or outside the cells, confocal microscopy analyses were performed. Fibroblasts were incubated with FITC-labeled NMPs (0.25 mg/mL) for 4, 24, or 48 h. After incubation cells were stained with a red fluorescent marker WGA for highlighting plasma and Golgi/ER membrane structures. Results are shown in Figure 9, Figure 10, Figure 11 and Figure 12, where numerous NMPs were visualized on treated cells by confocal microscopy imaging prolonging the incubation time up to 48 h (TOP panels). All prepared NMPs appear localized (yellow signal) with the cell membrane marker (red signal) after 48 h incubation. It is worth noting that NMPs were found to non-specifically adsorb different dyes, hence the colocalization study could be affected by this interference. Although the internalization process cannot be excluded for certain, the acquired confocal microscope micrographs convey that the NMPs are adherent to the cell membrane and seem to be localized on the outside of it when examining all three projections (Figure 9, Figure 10, Figure 11 and Figure 12, BOTTOM panel).

Typically, microparticles with a diameter longer than 0.5 µm have been known to enter cells via phagocytosis pathways [21]. Generally, the phagocytosis pathway is restricted to specialized phagocytes, such as macrophages, monocytes, and polymorphonuclear granulocytes. However, it has been shown that some cell types, such as fibroblasts, epithelial cells, and endothelial cells, can also internalize large particles [22]. From confocal microscopy images, we can deduce that NMPs instead seem not to be internalized and dock on the outside of the cell membrane. Overall, these studies show the effective interaction and internalization of NMP with fibroblast without compromising their proliferation. However, further studies will be needed to clarify the precise uptake mechanism of the NMP as well as the particles’ interactions with other cell types.

## 4. Conclusions

In this work, we demonstrated the general use of one-pot self-precipitation/cross-linking technique to prepare highly porous polysaccharide particles based on polysaccharides, namely dextran, carboxymethyl dextran, alginate, and (low molecular weight) hyaluronic acid. Our methodology involves the cross-linking agent precipitation to form crystalline domains embedding the polysaccharides chains which act as a “glue” between the crystalline domains. The study proves the robustness and versatility of this fabrication method.

The efficient loading of BSA and lysozyme into the NMP was shown along with the sustained release of the two proteins. The dependence of the adsorption-release performance on pH was demonstrated as well, which bears implications in medical applications and represents a possible way of tweaking the release profile. Finally, cell interaction was investigated to prove that NMPs are not cytotoxic but markedly biocompatible. Overall, this work highlights that this methodology and nanostructured microsystems could offer an innovative platform for biomedical and clinical applications.

## Figures and Tables

**Table 1:** Nanoporous microsponge particle (NMP) microstructures for different polysaccharides and for the two protocols (DMSO vs. H2O solvent of cross-linker).

Polysaccharide	Structure	DMSO-Protocol		H_2_O-Protocol	
		Confocal	SEM	Confocal	SEM
**CM-Dextran**	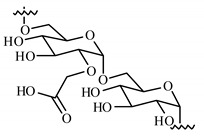	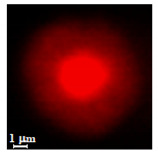	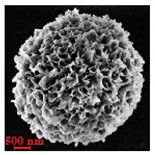	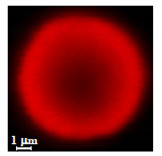	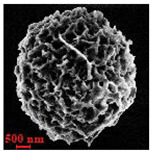
**Hyaluronic acid**	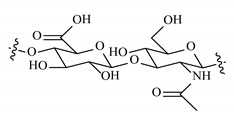	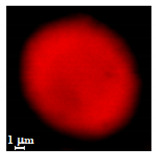	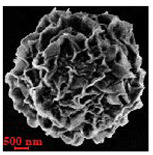	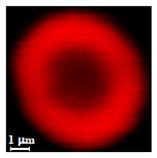	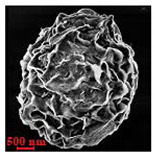
**Alginate**	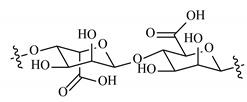	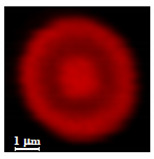	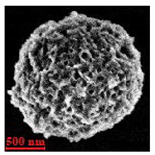	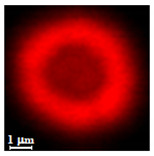	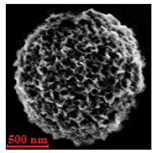
**Dextran**	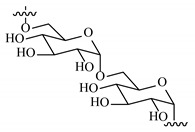	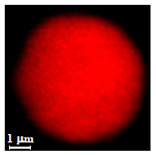	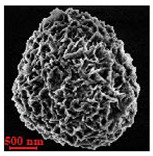	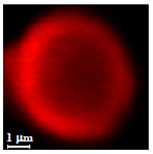	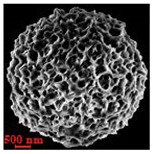

**Table 2:** A description of the reaction conditions of the 8 batches vs. yields and structural properties of NMP (diameter, pore size, swelling ratio).

Polysaccharide	Solvent for CL		Average Yield (%)	NMP Diameter			NMP Pores Diameter
				Dry (SEM)	Swollen	Swelling Ratio	
CM-Dextran	DMSO	→	97	3.5 ± 0.8 µm	9.8 ± 1.7 µm	2.8	0.10 ± 0.04 µm
	H_2_O		94	5.0 ± 0.1 µm	9.6 ± 1.8 µm	1.9	0.13 ± 0.05 µm
Hyaluronic Acid	DMSO		98	4.0 ± 0.8 µm	11.2 ± 1.8 µm	2.8	0.16 ± 0.06 µm
	H_2_O		98	3.4 ± 0.7 µm	7.3 ± 1.4 µm	2.1	0.14 ± 0.05 µm
Alginate	DMSO		98	1.4 ± 0.4 µm	5.2 ± 1.6 µm	3.5	0.06 ± 0.02 µm
	H_2_O		98	1.2 ± 0.5 µm	6.3 ± 1.0 µm	5.3	0.08 ± 0.03 µm
Dextran	DMSO		96	2.4 ± 0.8 µm	7.4 ± 1.8 µm	3.0	0.11 ± 0.04 µm
	H_2_O		96	4.8 ± 1.0 µm	7.5 ± 1.4 µm	1.6	0.09 ± 0.04 µm

**Table 3:** Adsorption of Lysozyme and BSA in NMPs (*d: Dimethyl Sulphoxide; w: Water*).

Polysaccharide	Adsorbed Lysozyme Quantity (mg) after 24 h		Adsorbed BSA Quantity (mg) after 24 h	
	d-NMP	w-NMP	d-NMP	w-NMP
CM-Dextran	1.099	0.441	0.999	0.850
Hyaluronic acid	1.155	0.553	0.814	0.894
Alginate	1.252	0.402	0.944	0.886
Dextran	1.225	0.432	0.868	0.913

## Supplementary Materials

The following are available online at https://www.mdpi.com/2079-4991/10/6/1075/s1, Figure S1–S4: NMR Spectra, Figure S5: DTT accelerated NMP degradation, Table S1: DMSO-Particles degradation by DTT, Table S2: H_2_O-Particles degradation by DTT.
