# Supplementary material for: Nanoporous Microsponge Particles (NMP) of Polysaccharides as Universal Carriers for Biomolecules Delivery
Source: Nanomaterials (Basel). 2020 May 31;10(6):1075. doi: 10.3390/nano10061075 (PMC7353405; doi:10.3390/nano10061075)
Supplement: Supplementary file 1 [file nanomaterials-10-01075-s001.pdf]

## Supplementary Materials

# Nanoporous Microsponge Particles (NMP) of Polysaccharides as Universal Carriers for Biomolecules Delivery

**Maria Federica Caso [1,†], Felicia Carotenuto [2,3,†], Paolo Di Nardo [2,3,4], Alberto Migliore [5], Ana Aguilera [6], Cruz Matilde Lopez [6], Mariano Venanzi [7], Francesca Cavalieri [7,*] and Antonio Rinaldi [1,8,*]**

1 NANOFABER srl, 00123 Rome, Italy; maria.federica.caso@gmail.com
2 Center of Regenerative Medicine, University of Rome "Tor Vergata", 00133 Rome, Italy; carotenuto@med.uniroma2.it (F.C.); dinardo@uniroma2.it (P.D.N.)
3 Department Clinical Sciences and Translational Medicine, University of Rome "Tor Vergata", 00133 Rome, Italy
4 I.M. Sechenov First Moscow State Medical University, 119991 Moscow, Russia
5 Rheumatology Department, San Pietro Hospital Fatebenefratelli, 00189 Rome, Italy; migliore.alberto60@gmail.com
6 Center for Genetic Engineering and Biotechnology, 10600 Havana, Cuba; ana.aguilera@cigb.edu.cu (A.A.); matilde.lopez@cigb.edu.cu (C.M.L.)
7 Department of Chemical Science and Technologies, University of Rome "Tor Vergata", 00133 Rome, Italy; venanzi@uniroma2.it
8 PROMAS-MATPRO Laboratory, Sustainability Department, ENEA, 00123, Rome, Italy
* Correspondence: francesca.cavalieri@uniroma2.it (F.C.); antonio.rinaldi@nanofaber.com (A.R.); +39-06-7259-4461 (F.C.); Tel.: +39-06-3048-3393 (A.R)
† These authors contributed equally to this work.

Received: 9 May 2020; Accepted: 23 May 2020; Published: date

## 1. Synthesis and characterization of cross-linker

Cross-linker (CL) dissolved in DMSO was examined by NMR. Spectra were recorded on NMR spectrometer operating at 400 MHz (Bruker DRX, Bruker AVANCE) and mass spectrometry.

*Purified product.*

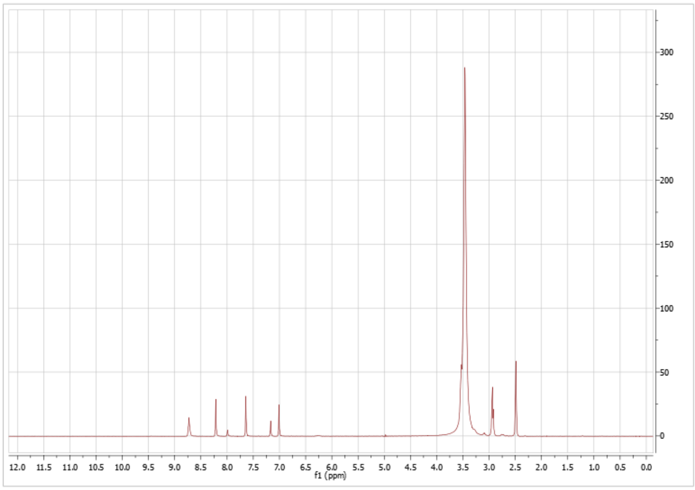


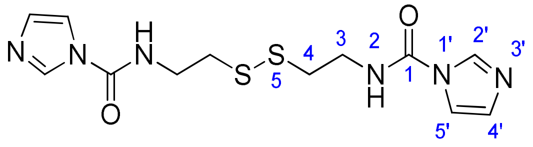


**Figure S1.** Spectrum $^{1}$H, 400 MHz, DMSO-*d*$_6$. $\delta$: 2.49 (t, *J* = 1.4 Hz, 4H, H-4), 2.93 (t, *J* = 8.8 Hz, 4H, H-3), 7.01 (br s, 2H, H-5'), 7.17 (br s, 1H, ?), 7.64 (br s, 2H, H-4'), 7.99 (br s, ?), 8.22 (br s, 2H, H-2'), 8.73 (t, *J* = 4.8 Hz, 2H, H-2). NOTE: Peaks at 7.17, 7.99 and 7.01 do not belong to product but to imidazole, byproduct obtained with degradation in water of CDI.

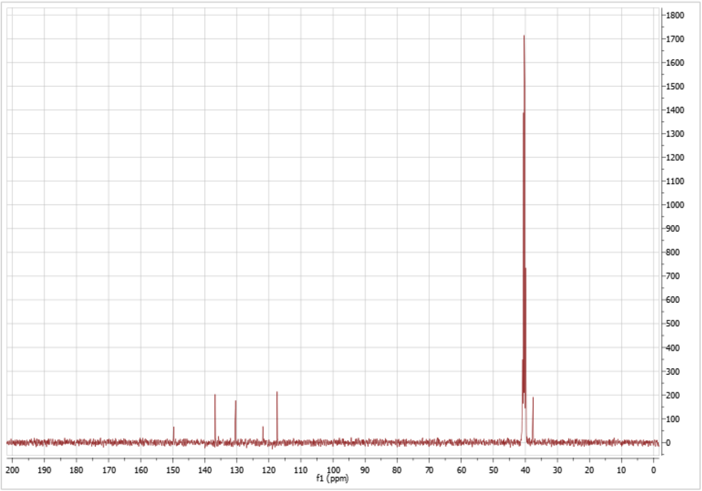


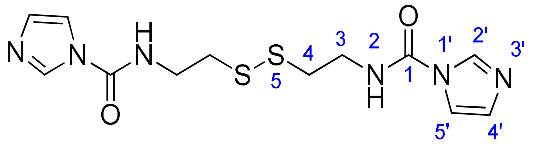


**Figure S2.** Spectrum 13C, 100 MHz, DMSO-d$_6$. $\delta$: 37.6 (C-4), 41.0 (C-3), 117.4 (C-5'), 121.9 (starting product), 130.4 (C-4'), 136.8 (C-2'), 149.7 (C-1). NOTE: Peak of C-3 is under DMSO signal, while 121 and 136 belong to imidazole.

*Crude product*

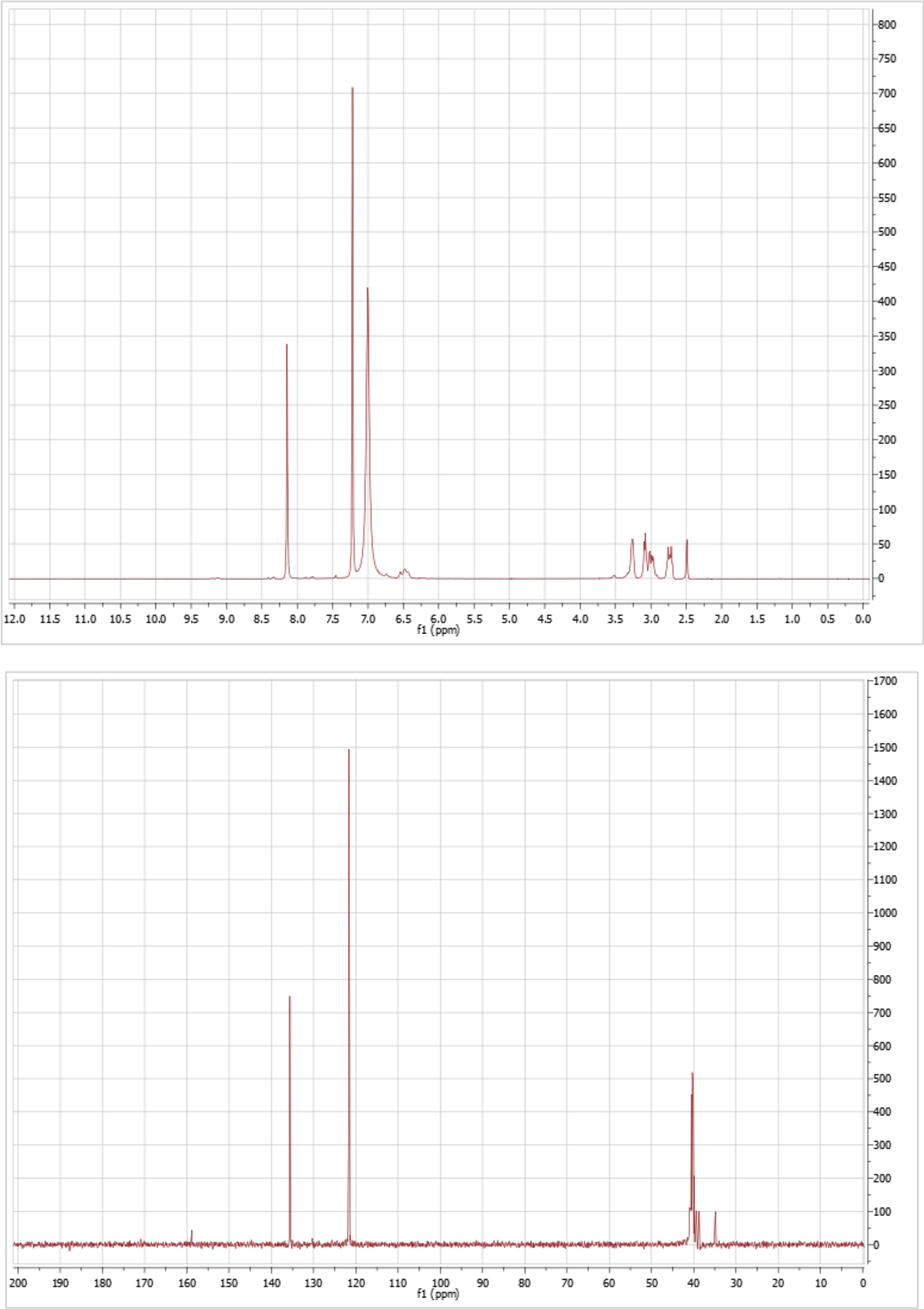


**Figure S3.** Peaks from 30 to 40 ppm indicate the presence of the following molecules (NOTE: Peaks 7.01, 7.22, 8.14 in 1H and 121.7, 135.6 in 13C belong to imidazole).

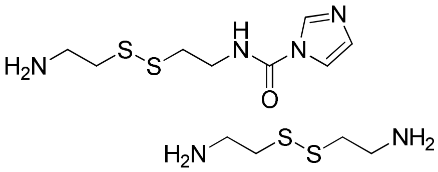

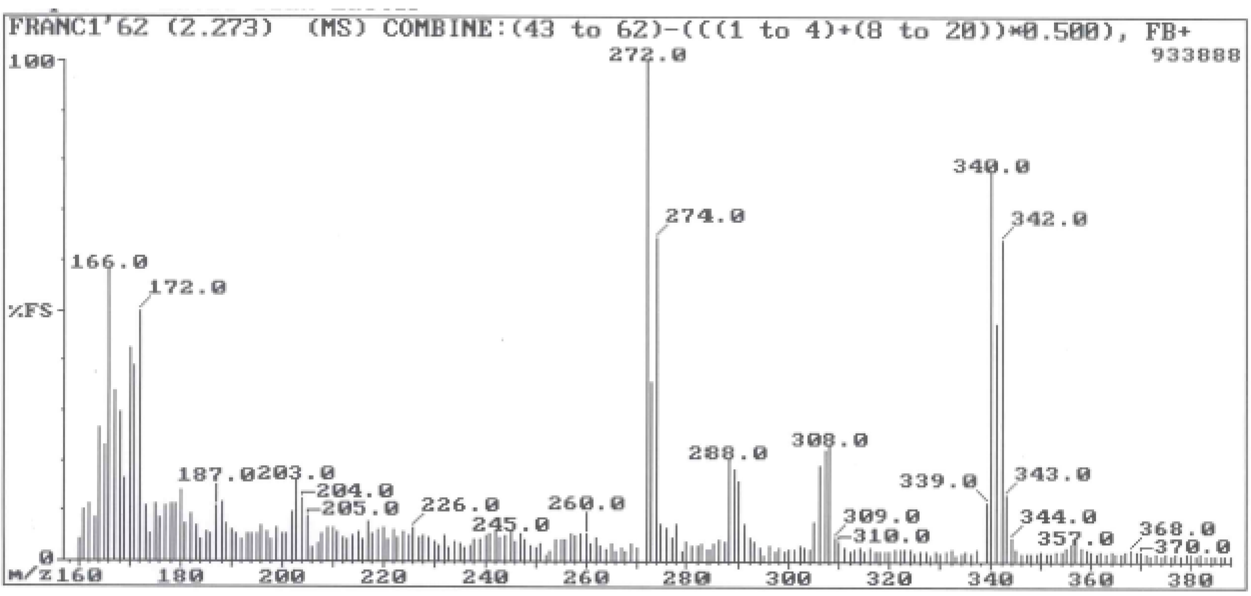


**Figure S4.** FAB mass spectra were obtained with a VG-Quattro spectrometer in the positive-ion mode by 3-nitrobenzylalcohol (peak 306, Sigma Aldrich) as the matrix. Resulting peaks derived from fragments:

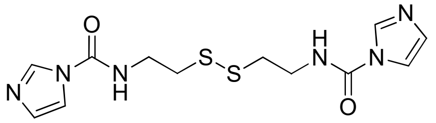

Molecular Weight: 340,42

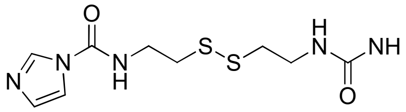

Molecular Weight: 288,37

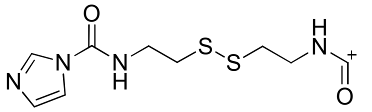

Molecular Weight: 273,35

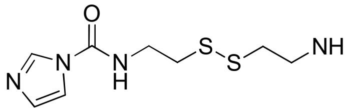

Molecular Weight: 245,35

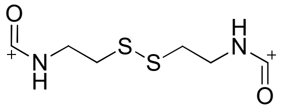

Molecular Weight: 206,28

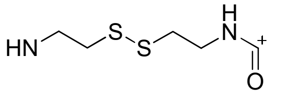

Molecular Weight: 178,28

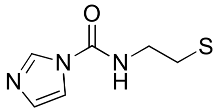

Molecular Weight: 170,21

## 2. DTT Degradation Kinetics of NMPs

50 μL of suspensions containing labeled microparticles (carbohydrate concentrations: 1.51 g/L) were added to 1 mL of a solution 0.01 M of DL-Dithiothreitol (DTT, Sigma-Aldrich, 99%) in PBS. At variable time intervals, the suspension was centrifuged and the supernatant was analyzed with UV-vis spectra. The degradation study was carried out for 24 h, when the degradation resulted complete, as shown in Tables S1,2.

**Table S1.** DMSO-Particles degradation by DTT.

| Polymer | Yield 1h (%) | Yield 2h (%) | Yield 3h (%) | Yield 4h (%) | Yield 6h (%) | Yield 9h (%) | Yield 24h (%) |
|---|---|---|---|---|---|---|---|
| CM-Dextran | 31.9 | 59.8 | 66.7 | 72.4 | 79.3 | 89.7 | 99.3 |
| HA low MW | 21.0 | 60.9 | 70.7 | 81.5 | 88.4 | 92.5 | 98.8 |
| Alginate | 25.0 | 53.2 | 68.5 | 81.8 | 85.1 | 91.2 | 99.3 |
| Dextran | 40.0 | 60.5 | 66.0 | 76.6 | 85.3 | 88.3 | 99.6 |

**Table S2.** $H_2O$-Particles degradation by DTT.

| Polymer | Yield 1 h (%) | Yield 2 h (%) | Yield 3 h (%) | Yield 4 h (%) | Yield 6 h (%) | Yield 9 h (%) | Yield 24 h (%) |
|---|---|---|---|---|---|---|---|
| CM-Dextran | 27.1 | 51.3 | 72.3 | 84.8 | 94.8 | 97.9 | 99.8 |
| HA low MW | 10.2 | 40.4 | 63.9 | 77.6 | 88.8 | 95.3 | 99.2 |
| Alginate | 11.1 | 42.9 | 63.7 | 71.4 | 89.1 | 92.6 | 99.7 |
| Dextran | 27.5 | 54.1 | 75.0 | 85.2 | 96.4 | 99.9 | 100.0 |

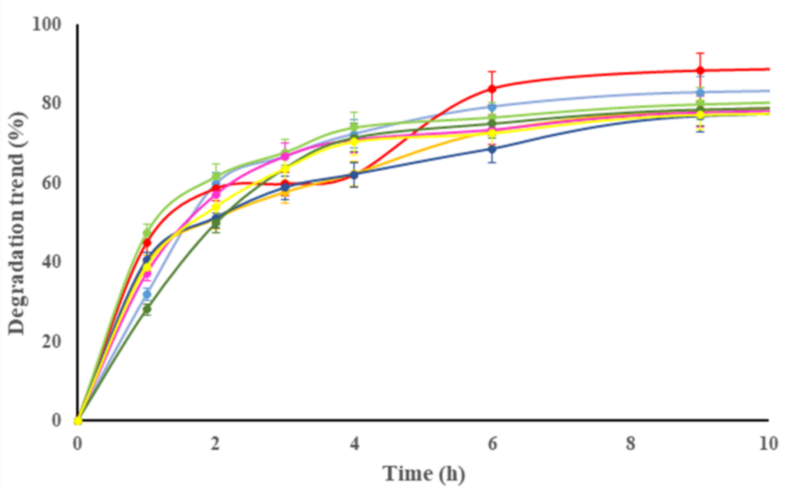


**Figure S5.** Particles degradation trend. Light blue: CM-dextran DMSO-particles; red: hyaluronic acid DMSO-particles; light green: alginate DMSO-particles; orange: dextran DMSO-particles; blue: CM-dextran $H_2O$-particles; pink: hyaluronic acid $H_2O$-particles; dark green: alginate $H_2O$-particles; yellow: dextran $H_2O$-particles.
